# Genome Announcement: Draft Genome Assembly of *Heterodera humuli* Generated Using Long-Read Sequencing

**DOI:** 10.2478/jofnem-2024-0029

**Published:** 2024-08-28

**Authors:** Lester A. Núñez-Rodríguez, Catherine L. Wram, Cedar Hesse, Inga A. Zasada

**Affiliations:** Department of Botany and Plant Pathology, Oregon State University, Corvallis, OR 97331; USDA-ARS Mycology and Nematology Genetic Diversity and Biology Lab, Beltsville, MD 20705; USDA-ARS Horticultural Crops Production and Genetic Improvement Research Unit, Corvallis, OR 97331; USDA-ARS Horticultural Crops Disease and Pest Management Research Unit, Corvallis, OR 97331

**Keywords:** cyst nematodes, *Heterodera humuli*, hop, long reads, ultra-low DNA input

## Abstract

The hop cyst nematode, *Heterodera humuli*, is the most common plant-parasitic nematode associated with hop worldwide. This study reports the draft genome of *H. humuli* generated on the PacBio Sequel IIe System with the ultra-low DNA input HiFi sequencing method, and the corresponding genome annotation. This genome resource will help further studies on *H. humuli* and other cyst nematodes.

Cyst nematodes are considered the second most important plant-parasitic nematodes (PPNs) worldwide, with *Globodera* and *Heterodera* as the most important genera (Jones et al., 2013). These PPNs are important not only for the extensive crop loss they cause but also for their ability to survive for long periods of time (> 20 years) in soil without a suitable host (Wright and Perry, 2006). The genus *Heterodera* is divided into nine groups based on morphological and molecular information: *Afenestrata, Avenae, Bifenestra, Cardiolata, Cyperi, Goettingiana, Humili, Sacchari,* and *Schachtii* (Handoo and Subbotin, 2018). The hop cyst nematode, *Heterodera humuli*, is the most common PPN associated with hop worldwide. *Heterodera humuli* is commonly found in Washington, Idaho, and Oregon, the most important states for hop production in the U.S. (Darling et al., 2023). Despite the importance of the pest, very little molecular data is available for *H. humuli*, including a lack of genomic or transcriptomic resources. Of the 82 described *Heterodera* spp. there are only three publicly available genomes, two from the *Schachtii* group (*H. glycines* and *H. schachtii*), and one from the *Goettingiana* group (*H. carotae*) (Masonbrink et al., 2019; Siddique et al., 2022; Wram et al., 2022). Generating a high-quality genome of *H. humuli* will provide an important resource for research regarding the management of *H. humuli* and for a wider range of cyst nematode investigations.

In 2022, a hopyard in Salem, Oregon was sampled for *H. humuli*. Briefly, soil was collected from seven plots (five soil cores/plot) using a 2.5-cm-diameter soil probe with a probe length of 30 cm (JMC, Newton, IA). The soil was mixed and placed on an aluminum tray to dry it for at least one week in a greenhouse at the Nematology Lab at USDA-ARS-HCDPMRU in Corvallis, Oregon. Cysts were extracted from dried soil using the USDA-cyst extractor (Zasada et al., 2019). Cysts (n = 115) were handpicked, placed in a petri dish, and cut to release encysted eggs. The eggs were cleaned and transferred into a 1.5 ml Eppendorf tube then filled with water to a final volume of 0.5 ml. The total number of nematodes (eggs and second-stage juveniles) was determined by counting a subsample of 50 µl. Excess water was subsequently removed by centrifugation and aspiration to a final volume of approximately 50 µl. Nematodes were shipped overnight to the Roy J. Carver Biotechnology Center, University of Illinois at Urbana-Champaigne, Illinois on dry ice for DNA extraction and sequencing. DNA extraction was performed as in Arora et al. (2023). Briefly, high molecular weight DNA was extracted from a total of 10,200 nematodes with the MagAttract HMW DNA kit (Qiagen, Hilden, Germany). The High-Sensitivity dsDNA kit (ThermoFisher Scientific, Waltham, MA) was used to determine DNA concentration using a Qubit (Thermo Fisher Scientific). DNA integrity was assessed using a Femto Pulse system (Agilent, Santa Clara, CA).

Purified genomic DNA (5 ng) was sheared with Megaruptor 3 (Diagenode, Denville, NJ), amplified using the PacBio Ultra-Low DNA input kit following the company’s protocol (Pacific Biosciences, Menlo Park, CA), and converted into a PacBio library with the SMRTbell Express Template Prep kit version 2.0 (PacBio Biosciences). Qubit was used for library quantification and Femto Pulse for fragment size distribution. Sequencing was carried out on 1 SMRT cell 8 M on a PacBio Sequel IIe system using the circular consensus sequencing (CCS) mode and a 30h movie time with default parameters (three minimum passes and a minimum accuracy of 99%). The sequencing yielded 14.9 GB of CCS data. The total number of reads was 2,800,608 with a mean read length of 5,327 bp.

PacBio HiFi reads were filtered with the GNU Awk program v.4.0.2 (http://www.gnu.org/licenses/) and reads less than 3 kb were removed leaving 1,791,046 reads for assembly. Hifiasm v.0.16.1-r375 (Cheng et al., 2021) with default parameters was used for genome assembly. Blobtools v.1.0 (Laetsch and Blaxter, 2017) with default parameters used to visualize *de novo* assembled contigs based on the read coverage and GC content. To determine coverage, raw data was mapped to the de novo assembly using minimap2 with default parameters (Li, 2018). A phylum identification was given to each contig in the assembly based on BLAST similarity (E-value < 10e^−25^) to sequences in the NCBI “nt” database or other PPN genomes (*Heterodera carotae* [NCBI BioProject:PRJNA774818], *H. glycines* [NCBI BioProject:PRJNA381081], *H. schachtti* [NCBI BioProject:PRJNA722882], *Meloidogyne incognita* [ENA Project:PRJEB8714], *Radopholus similis* [NCBI BioProject:PRJNA541590], *Globodera rostochiensis* [ENA Project: PRJEB13504], and *Globodera pallida* [ENA Project: PRJEB123]). Reads belonging to contigs with >10X coverage and identified as Nematoda or with no assigned identity (no-hit) were used to reassemble the *H. humuli* genome using the Hifiasm v.0.16.1-r375 algorithm. Blobtools v.1.0 was again used to visualize the re-assembly based on the read coverage and GC content as described previously. After the filtered assembly was complete, contigs identified as belonging to the phylum Nematoda or with no identity (1,487 contigs out of 1,489 contigs) were retained to make the final genome assembly for *H. humuli*. The Blobtools visualizations of the meta and final assemblies are in Supplemental Figs. 1 and 2, respectively. Genome statistics (Table 1) were obtained using QUAST 5.2.0 default parameters (Gurevich et al., 2013).

**Table 1.** Comparison of genome assembly statistics of *Heterodera humuli* and three other *Heterodera* species.

**Type of analysis**	**Parameter**	***H. carotae* (PRJNA774818)^x^**	***H. glycines* (PRJNA381081)^x^**	***H. humuli* (PRJNA1048471)^x^**	***H. schachtii* (PRJNA767548)^x^**
Genome statistics	Total Number of bp	95,115,141	157,978,452	90,806,450	190,153,211
	Number of Contigs	17,835	9	1,487	705
	Largest contig	113,425	23,985,585	568,746	4,323,191
	Contigs ≥ 25000	699	9	871	609
	Contigs ≥ 50000	103	9	559	531
	Average GC%	39.39	36.66	36.11	32.36
	Contig N50	13,935	17,907,690	111,383	500,954
	Contig L50	1,836	4	231	86
	Number of N’s per 100 kbp	0	1,062.01	0	2,253.83
	Predicted number coding genes	17,037	22,465	15,428	29,851
	Predicted number of transcripts	17,322	29,933	16,848	31,564

Predicted Protein BUSCO Analysis (Nematoda_ob10 – 3,131 genes)	Complete BUSCOs (C) (%)	1,301 (41.5%)	1,884 (60.2%)	2,030 (64.9%)	2,080 (66.4%)
	Complete BUSCOs and single-copy BUSCOs (S) (%)	1,237 (39.5%)	1,612 (51.5%)	1,590 (50.8%)	1,675 (53.5%)
	Complete and duplicated BUSCOs (D) (%)	64 (2%)	272 (8.7%)	440 (14.1%)	405 (12.9%)
	Fragmented BUSCOs (F) (%)	124 (4%)	47 (1.5%)	66 (2.1%)	69 (2.2%)
	Missing BUSCOs (M) (%)	1,706 (54.5%)	1,200 (38.3%)	1,035 (33%)	982 (31.4%)

Predicted Protein BUSCO Analysis (Eukaryota_ odb10 - 255 genes)	C (%)	160 (62.8%)	199 (78%)	221 (86.6%)	224 (87.8%)
	S (%)	156 (61.2%)	176 (69%)	174 (68.2%)	200 (78.4%)
	D (%)	4 (1.6%)	23 (9%)	47 (18.4%)	24 (9.4%)
	F (%)	52 (20.4%)	20 (7.8%)	13 (5.1%)	15 (5.9%)
	M (%)	43 (16.8%)	36 (14.2%)	21 (8.3%)	16 (6.3%)

x NCBI GenBank BioProject number.

The objective of this genome sequencing effort was to provide high quality reference data to expand the publicly available genomic resources for cyst nematodes. The assembled genome size of *H. humuli* is 90,806,450 bp in a total of 1,487 contigs (Table 1) with an average 112X coverage. The genome provided in this study is smaller than the other available *Heterodera* spp. genomes, up to ~ 100 Mb less when compared to the *H. schachtii* genome (Table 1). The potential genome size and repeat length for *H. humuli* are estimated to be 90,077,635 and 63,372,724 bp, respectively, by using reads > 3 kb as input and a kmer size of 21 in GenomeScope (Vurture et al., 2017). The assembly has a GC content of 36.11% and a N50 of 111,383 bp (Table 1).

Completeness of the assembly of *H. humuli* and the genomes of the three *Heterodera* spp. mentioned above was determined with BUSCO v5.4.5 using the eukaryota_odb10 and nematoda_odb10 databases (Manni et al., 2021) (Supplementary Fig. 3). *Heterodera humuli* had the second and third most complete number of BUSCO genes when using the eukaryota_odb10 and nematoda_odb10 databases, respectively (Supplementary Fig. 3).

To annotate the genome of *H. humuli*, highly repetitive regions were identified and masked using RepeatModeler v 2.0.2 and RepeatMasker v 4.1.0, respectively, using default parameters (Smit and Hubley, 2015a; Smit and Hubley, 2015b). Predicted proteins from the *H. glycines* genome (Masonbrink et al., 2019) were used as protein hints to annotate the masked genome with BRAKER3 v 3.0.3 using default parameters (Lomsadze et al., 2005; Stanke et al., 2006; Gotoh, 2008; Stanke et al., 2008; Iwata and Gotoh, 2012; Buchfink et al., 2015; Hoff et al., 2016; Hoff et al., 2019; Bruna et al., 2020; Bruna et al., 2021). In general, *H. humuli* has a smaller number of predicted number of transcripts compared to the other *Heterodera* species included in this analysis. When compared to *H. schachtii*, *H. humuli* has 14,716 fewer transcripts (Table 1). The BUSCO analysis was also performed on the predicted proteins from all *Heteredora* spp. included in this study using both the nematoda_odb10 and eukaryota_odb10 databases. *Heterodera humuli* had the second highest complete BUSCO score with both the nematoda_odb10 (64.9 %) and eukaryota_odb10 (86.6 %) databases (Table 1).

The partial mitochondrial *cox1* gene was retrieved from the *H. humuli* assembly using usearch11 (Edgar, 2010) with the *H. humili* accession number ON007077 from NCBI. Other *cox1* sequences from *Heterodera* spp. were retrieved from NCBI, along with outgroup *Rotylenchus* spp., to perform a phylogenetic analysis. Sequences were aligned with the Clustal W algorithm with default parameters (Thompson et al., 1994) and trimmed using BioEdit v.7.0.5.3 (Hall, 1999). The best substitution model was determined using jModelTest 2.1.10 v20160303 with default parameters (Darriba et al., 2012) and a Bayesian phylogenetic inference was performed using MrBayes v3.2 (Ronquist et al., 2012) at the CyberInfrastructure for Phylogenetic Research (CIPRES) Science Gateway (Miller et al., 2010) with default parameters. Subsequently, FigTree v1.4.3 (Rambaut, 2016) was used to visualize the resulting phylogenetic tree. The phylogenetic analysis placed the *cox1* sequence from this *H. humuli* assembly (coded as ptg000050c_HCN) in a clade with other *H. humuli* sequences with a posterior probability value of 100% (Supplementary Fig. 4). Thus, confirming the identity of the species.

Genome assembly, annotation, and raw data for *H. humuli* can be found at NCBI under the BioProject number PRJNA1048471 (BioSample number SAMN38640877). This genome of *H. humili* generated using long reads offers a valuable genetic resource for future research efforts on the biology of *H. humuli*, discovery of novel effectors, genomic comparison among cyst forming nematodes, and many other aspects.

## Figures and Tables

**Figure S1.**
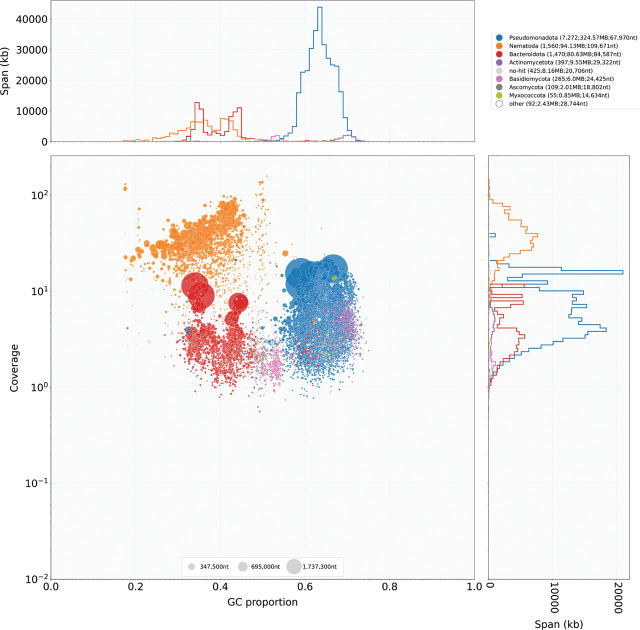
Visualization of the meta-assembly of *Heterodera humuli* genome. The Hifiasm v.0.16.1-r375 algorithm was used to assemble the reads > 3 kb from the PacBio Sequel IIe system. The Blobplot (Laetsch and Blaxter, 2017) shows assembled contigs as circles with placement of circles on the x-axis representing the proportion of GC bases and the position on the y-axis representing the coverage of the contig with the raw data.

**Figure S2.**
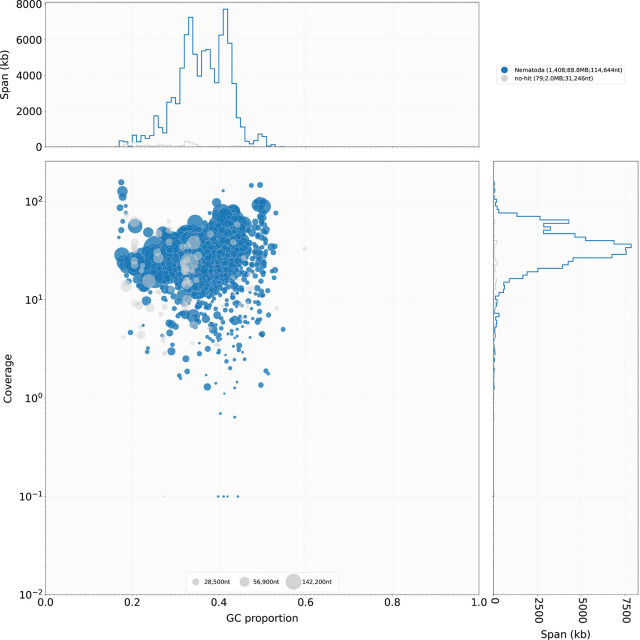
Visualization of the final assembly of *Heterodera humuli* using reads from the PacBio Sequel IIe system. The Hifiasm v.0.16.1-r375 algorithm was used to assemble the reads. The blobplot (Laetsch and Blaxter, 2017) shows assembled contigs as circles with placement of circles on the x-axis representing the proportion of GC bases and the position on the y-axis representing the coverage of the contig with the raw data.

**Figure S3.**
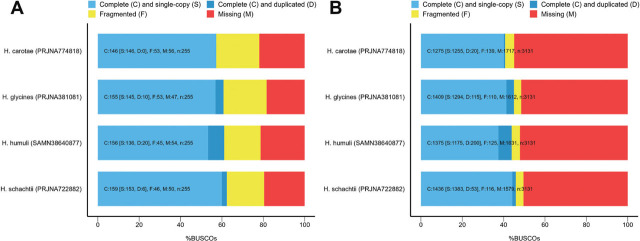
BUSCO assessment results of *Heterodera* species (NCBI GenBank BioProject number) using both databases, A) eukaryota_odb10 and B) nematoda_odb10.

**Figure S4.**
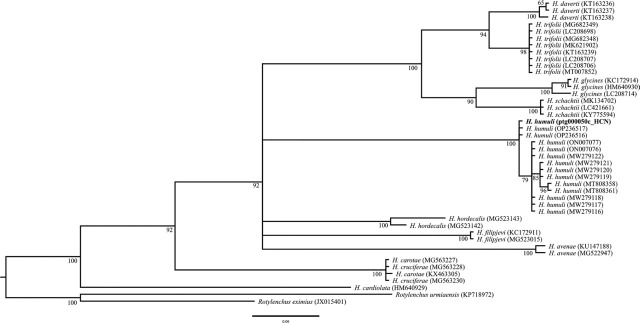
Phylogenetic relationships between *Heterodera* species as inferred from Bayesian analysis of the partial *cox1* gene sequences under the GTR + I + G model. Posterior probability values are given for appropriate clades. The bold name represents the *H. humuli* sequence derived from this study. Sequences were retrieved from GenBank, aligned, and trimmed with BioEdit v.7.0.5.3 (Hall, 1999). The best substitution model was determined using jModelTest 2.1.10 v20160303 (Darriba et al., 2012) and the Bayesian analysis was performed using MrBayes v3.2 (Ronquist et al., 2012) at the CIPRES Science Gateway. Finally, FigTree v1.4.3 (Rambaut, 2016) was used to visualize the resulting phylogenetic tree.
